# Treatment of relapsed or refractory classical Hodgkin lymphoma with the anti-PD-1, tislelizumab: results of a phase 2, single-arm, multicenter study

**DOI:** 10.1038/s41375-019-0545-2

**Published:** 2019-09-13

**Authors:** Yuqin Song, Quanli Gao, Huilai Zhang, Lei Fan, Jianfeng Zhou, Dehui Zou, Wei Li, Haiyan Yang, Ting Liu, Quanshun Wang, Fangfang Lv, Haiyi Guo, Liudi Yang, Rebecca Elstrom, Jane Huang, William Novotny, Vivian Wei, Jun Zhu

**Affiliations:** 1 Key Laboratory of Carcinogenesis and Translational Research (Ministry of Education), Department of Lymphoma, Peking University Cancer Hospital and Institute, Beijing, China; 2 Department of Immunotherapy, Affiliated Cancer Hospital of Zhengzhou University, Henan Cancer Hospital, Zhengzhou, China; 3 Tianjin Medical University Cancer Institute and Hospital, National Clinical Research Center for Cancer, Key Laboratory of Cancer Prevention and Therapy, Tianjin’s Clinical Research Center for Cancer, Tianjin, China; 4 Department of Hematology, the First Affiliated Hospital of Nanjing Medical University, Jiangsu Province Hospital, Collaborative Innovation Center for Cancer Personalized Medicine, Nanjing, China; 5 Department of Hematology, Tongji Hospital, Tongji Medical College, Wuhan, China; 6 State Key Laboratory of Experimental Hematology, Institute of Hematology and Blood Diseases Hospital, Chinese Academy of Medical Sciences and Peking Union Medical College, Tianjin, China; 7 Department of Hematology, Cancer Center, The First Hospital of Jilin University, Changchun, China; 8 Department of Oncology, Zhejiang Cancer Hospital, Hangzhou, China; 9 Department of Hematology, West China Hospital of Sichuan University, Chengdu, China; 10 Department of Hematology, Chinese PLA General Hospital, Beijing, China; 11 Department of Medical Oncology, Fudan University Shanghai Cancer Center, Shanghai, China; 12 BeiGene (Beijing) Co., Ltd., Beijing, China; 13 BeiGene USA, Inc., San Mateo, CA USA

**Keywords:** Hodgkin lymphoma, Phase II trials

## Abstract

Prognosis is poor for patients with relapsed/refractory (R/R) classical Hodgkin lymphoma (cHL) after failure of or who are ineligible for autologous stem cell transplant. We evaluated the efficacy and safety of tislelizumab, an investigational anti-PD-1 monoclonal antibody, in phase 2, single-arm study in Chinese patients with R/R cHL. The primary endpoint was overall response rate as assessed by an independent review committee, according to the Lugano 2014 Classification. Seventy patients were enrolled in the study and received at least one dose of tislelizumab. After median follow-up of 9.8 months, 61 (87.1%) patients achieved an objective response, with 44 (62.9%) achieving a complete response (CR). The estimated 9-month progression-free survival rate was 74.5%. Most common grade ≥3 adverse events (AEs) were upper respiratory tract infection and pneumonitis. Infusion-related reactions occurred in 27 (38.6%) patients, and 27 patients (38.6%) experienced an immune-related AE, the most common of which was thyroid dysfunction. Eleven (15.7%) patients experienced at least one treatment-emergent AE leading to dose interruption or delay. No deaths occurred due to AEs. Treatment of patients with R/R cHL with tislelizumab was generally well tolerated and resulted in high overall response and CR rates, potentially translating into more durable responses for these patients.

## Introduction

Classical Hodgkin lymphoma (cHL) accounts for 95% of all HL, with similar epidemiologic features between China and western countries [1, 2]. A hallmark of cHL is the presence of rare, CD30-positive Reed–Sternberg cells surrounded by an ineffective inflammatory and immune-cell infiltrate [3]. Reed–Sternberg cells often exhibit copy number alterations of programmed death ligand 1 and 2 (*PD-L1*/*L2*) on chromosome 9p24.1, resulting in overexpression of PD-1 ligands on tumor cells [4–6]. Engagement of PD-1 with its ligands results in downregulation of T-cell responses, thereby enabling tumor cells to evade immune surveillance. Alterations in *JAK2* copy number, also located on chromosome 9p24.1, lead to increased JAK–STAT signaling, further inducing PD-L1 overexpression [4]. Upregulation of PD-1 ligands in the cHL microenvironment allows malignant Reed–Sternberg cells to effectively evade immune surveillance and reveals a genetically determined predisposition to blockade of the PD-1/PD-L1 axis.

Nivolumab and pembrolizumab are fully human IgG4 monoclonal antibodies directed against PD-1. Both have demonstrated clinically meaningful activity in cHL patients following failure of high-dose chemotherapy and autologous hematopoietic stem cell transplant (HDT/ASCT), brentuximab vedotin [7], or both; however, only a minority of patients experience complete responses (CRs), and most patients experience disease progression within 18 months [8–11]. Tislelizumab is an investigational humanized IgG4 monoclonal antibody that has been shown to bind to the extracellular domain of human PD-1 with high specificity and affinity and block the binding of both PD-L1 and PD-L2. Tislelizumab was specifically engineered to minimize FcɤR binding on macrophages, thereby abrogating antibody-dependent phagocytosis, a potential mechanism of T-cell clearance and resistance to anti-PD-1 therapy [12]. Preclinical models demonstrate that this FcɤR modification leads to better antitumor activity in vivo, raising the potential that tislelizumab may induce deeper responses and longer duration of response (DOR). Pharmacokinetic analysis reveals a linear pharmacokinetic profile for tislelizumab, with a half-life of ~17 days [13]. In phase 1 studies, no clear dose-dependent relationship for either safety or efficacy was demonstrated at doses of 2 and 5 mg/kg administered every 2 or 3 weeks, and a uniform dose of 200 mg intravenously every 3 weeks was chosen for further investigation [13]. Clinical results from a first-in-human study (NCT02407990) demonstrated that tislelizumab was generally well tolerated and exhibited promising antitumor effects in patients with advanced solid tumors [14–16], results recapitulated in a separate phase 1 study in Chinese patients with advanced tumors treated with tislelizumab 200 mg every 3 weeks [17]. In light of tislelizumab’s preclinical data suggesting a potential advantage in durability of action compared with nivolumab and pembrolizumab, as well as its promising safety, pharmacokinetic, and preliminary efficacy profiles, we undertook the current phase 2 study to investigate tislelizumab in patients with relapsed/refractory (R/R) cHL (NCT03209973).

## Subjects and methods

### Patients

Patients were enrolled from 11 sites in China between April 21 and November 22, 2017. Eligible patients had cHL with measurable disease that was histologically confirmed by central pathologic review. Patients must have had relapsed or refractory cHL and have met one of the following criteria: (1) failed to achieve a response or progressed after ASCT or (2) received ≥2 prior systemic chemotherapy regimens for cHL and were considered ineligible for ASCT; the reason for ASCT ineligibility was required to be reported. Other eligibility criteria included age ≥ 18 years, Eastern Cooperative Oncology Group performance status of 0 or 1, and adequate organ function. An absolute neutrophil count of ≥1.5 × 10^9^/L and a platelet count of ≥75 × 10^9^/L as well as a hemoglobin concentration ≥8 g/dL (≥ 5 mmol/L) were required. Patients were excluded if they had known central nervous system lymphoma; prior exposure to a PD-1- or PD-L1-targeted agent; a history of allogeneic HSCT or ASCT within 100 days of the first dose of tislelizumab; clinically significant cardiovascular disease or myocardial infarction within the past 6 months; history of interstitial lung disease or noninfectious pneumonitis; known infection with HIV or serologic status reflecting active hepatitis B/C infection; or history of or active autoimmune disease at high risk for recurrence or exacerbation.

### Study design and treatment

This is an ongoing, phase 2, open-label, single-arm study of tislelizumab in Chinese patients with R/R cHL. All patients receive tislelizumab 200 mg administered intravenously every 3 weeks until disease progression, unacceptable toxicity, or study termination.

This study was designed and monitored in accordance with sponsor procedures and in compliance with the ethical principles of Good Clinical Practice, International Conference on Harmonization guidelines, the Declaration of Helsinki, and applicable local regulatory requirements. All patients provided written informed consent. The protocol, any amendments, and informed consent forms were approved by the institutional review boards/independent ethics committees.

### Assessments

The primary endpoint was the rate of overall response defined as either a partial response or CR as assessed by the independent review committee (IRC, Bioclinica, Princeton, NJ, USA) according to the Lugano classification [18] and based on fluorodeoxyglucose positron emission tomography (PET) scanning. Contrast-enhanced CT (or MRI) scans were performed at weeks 12, 18, 30, and 42 in the first year of study and every 15 weeks thereafter; PET scans were performed at weeks 12, 24, 42, and 57 and every 30 weeks thereafter. Patients continued tislelizumab if pseudoprogression was suspected, provided there was no concurrent clinical evidence of progression [19].

The secondary endpoints included DOR, time to response, progression-free survival (PFS), and safety. Overall survival was an exploratory endpoint. Adverse events (AEs) were coded using the Medical Dictionary for Regulatory Activities, version 19.1. AEs were graded for severity based on National Cancer Institute Common Toxicity Criteria, version 4.03.

Immune-related AEs (irAEs) were identified using a predefined Medical Dictionary for Regulatory Activities preferred term query followed by medical adjudication. Criteria used by the medical reviewers in assessing whether AEs were immune related included severity, time of onset in relationship to tislelizumab dosing, duration and treatment of the event, and potential alternative causes. Events assessed to be irAEs were categorized into major pathophysiologic subgroups, such as pneumonitis, colitis, hepatitis, etc.

### Statistical analysis

A binomial exact test indicated that a sample of 68 patients would provide 91% power to detect a difference in the overall response rate (ORR) of 35% (*H*_0_: ORR = 0.35; the minimal threshold for clinically meaningful benefit) versus 55% (*H*_*A*_: ORR = 0.55) at a one-sided alpha level of 0.025 and a 95% confidence interval of (0.425, 0.671), when the observed ORR was 55%. All results are presented as of July 23, 2018.

Efficacy and safety analyses included all patients with centrally confirmed cHL who received at least one dose of tislelizumab. Using the prespecified demographic and baseline disease characteristics, subgroup analyses were conducted for patients achieving a response.

Time-to-event, including DOR and PFS, were estimated using the Kaplan–Meier method with 95% confidence intervals calculated by the Brookmeyer and Crowley method [20]. Event-free rates at landmark time points were estimated by the Kaplan–Meier method with 95% confidence intervals estimated with Greenwood’s formula [21]. Patient follow-up was censored at the last adequate disease assessment before the initiation of subsequent anticancer therapy for PFS and DOR estimates. Patients without either a baseline or at least one postbaseline response assessment were censored on the first day of study treatment.

### Role of the funding source

BeiGene funded the study and provided the study drug. Investigators were responsible for designing the study protocol and statistical analysis plan together with BeiGene. The investigators and their respective research teams collected all the data, and BeiGene confirmed the accuracy of the data and compiled them for summation and analysis. Statistical analyses were performed by the biometrics group at BeiGene. The investigators have full access to the data and analyses. The study is being conducted under the supervision of an independent safety monitoring committee, the membership and procedures of which are outlined in a free-standing charter. Manuscript drafts were prepared by all the authors, with editorial assistance from a professional medical writer paid by BeiGene. All the authors vouch for the accuracy and completeness of the data reported and for the adherence of the study to the protocol, and all the authors made the decision to submit the manuscript for publication.

## Results

Seventy patients enrolled in the study and received at least one dose of tislelizumab. The median number of treatment cycles was 13 (range, 2–22), and the median relative treatment intensity was 100% (range, 62–101). All patients were evaluable for safety and efficacy. Baseline study population demographic and disease characteristics are summarized in Table 1. Sixty (85.7%) patients had advanced stage disease (Ann Arbor Stage IIb–IV). Thirteen patients (18.6%) had undergone prior ASCT; 57 (81.4%) patients were ineligible for ASCT, of whom 53 (93% of ASCT-ineligible patients) were ineligible due to having chemotherapy-resistant disease. Forty-five (52.3%) patients were refractory to their most recent therapy, and 25 (35.7%) patients had primary refractory disease (i.e., never achieved at least a partial response to any prior line of therapy). Four patients had received prior brentuximab vedotin, all on a clinical trial.

**Table 1:** Demographic and baseline disease characteristics

Characteristic	*N* = 70
Sex, *n* (%)	
Male	40 (57.1)
Female	30 (42.9)
Race, *n* (%)	
Chinese	70 (100)
Age, years	
Median (range)	32.5 (18–69)
≥65 years, *n* (%)	4 (5.7)
ECOG performance status, *n* (%)	
0	48 (68.6)
1	22 (31.4)
Median time from initial diagnosis, months	25.3
Advanced disease^a^, *n* (%)	60 (85.7)
Histologic subtype, *n* (%)	
Nodular sclerosis	42 (60)
Mixed cellularity	19 (27.1)
Lymphocyte rich	3 (4.3)
Unspecified	6 (8.6)
Bulky disease^b^, *n* (%)	8 (11.4)
Bone marrow involvement, *n* (%)	22 (31.4)
B-symptom(s), *n* (%)	26 (37.1)
Median (range) lines of prior therapy	3 (2–11)
Types of prior systemic therapy, *n* (%)	
Chemotherapy	70 (100)
ASCT	13 (18.6)
Immunotherapy^c^	15 (21.4)
Ineligible for prior ASCT^d^, *n* (%)	57 (81.4)
Patients with prior radiation therapy, *n* (%)	21 (30.0)
Refractory disease^e^, *n* (%)	45 (52.3)

^a^Advanced disease is defined as Ann Arbor Stage IIB, IIIA or B, Stage IIIE A or B, and Stage IV A or B

^b^Bulky disease defined as mediastinal mass ratio of 0.33 or size of any single node/nodal mass ≥ 10 cm in diameter

^c^Immunotherapy included brentuximab vedotin, rituximab, cytokine-induced killer cell transfusion, thalidomide, or lenalidomide

^d^Patients were ineligible for ASCT if they did not achieve at least a partial response to salvage chemotherapy, were ≥65 years of age, had contraindicating comorbidities, or due to the failure or inability to collect hematopoietic stem cells. All received ≥2 prior regimens

^e^Refractory disease was defined as the lack of at least a partial response to the last therapy before study entry, as assessed by the investigator

After a median follow-up of 9.79 months (range, 3.4–14.7), 17 patients (24.3%) had discontinued tislelizumab, and 53 were continuing treatment. Eleven patients discontinued study treatment due to disease progression and four patients discontinued due to AEs, including two patients with pneumonitis, one patient with organizing pneumonia, and one with focal segmental glomerulosclerosis. One patient discontinued the study after withdrawing consent, and one patient discontinued after becoming pregnant (Supplementary Table 1).

**Fig. 1:**
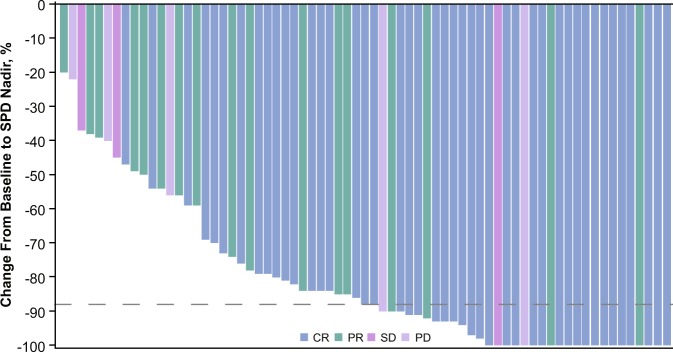
Maximum change from baseline in the SPD of target lesions for all patients. Percentage change in SPD is presented by best response achieved in each patient

**Fig. 2:**
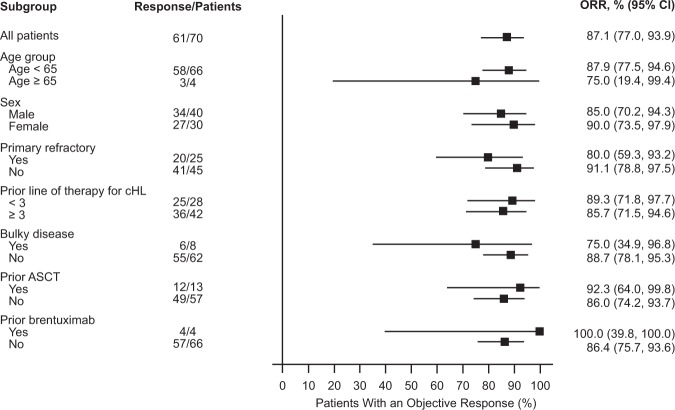
Overall response rate according to subgroup. This forest plot of data for 70 efficacy-evaluable patients shows the overall response rate according to defined demographic and baseline disease characteristics. The 95% confidence intervals are two-sided Clopper–Pearson estimations. For the category of baseline bone marrow involvement, “No” represents no involvement or not evaluable

All 70 patients were evaluable for efficacy. Of these, 61 (87.1%) achieved an objective response (95% CI: 77, 93.9; *P* *<* 0.0001 with respect to the null hypothesis of an overall response rate of 35%). Forty-four patients (62.9%) achieved a CR (Table 2, Supplementary Fig. 1). All patients experienced a decrease in disease burden (Fig. 1). Subgroup analysis revealed that responses to tislelizumab were generally consistent across all subgroups analyzed (Fig. 2). Of the 13 patients who had previously undergone ASCT, 12 (92.3%) achieved an objective response with nine (69.2%) achieving a CR, and of the four patients having previously received brentuximab vedotin, all achieved a CR. Of the 25 patients with primary refractory disease, 20 (80%) achieved an objective response including 13 (52%) CRs. The median time to response was 12 weeks. The investigator-assessed ORR and CR rates were 78.6 and 50%, respectively.

**Table 2:** Independent review committee-assessed efficacy outcomes

Efficacy variable	*N* = 70
Objective response, *n* (%)	
Complete	44 (62.9)
Partial	17 (24.3)
No response^a^	9 (12.8)
Overall (%)	87.1
95% CI for overall response rate	(77.0, 93.9)
P-value^b^	<0.0001
Time to response^c^, weeks	
Median (range)	12.0 (8.9–42.1)
DOR^c^, months	
Median^d^ (range)	NE (0.0+ to 10.3+)
95% CI	(NE, NE)
Event-free rates^c^ at 6 months (%)	84.1
95% CI	(70.3, 91.8)
Progression-free survival, months	
Median^d^ (range)	NE (2.6–13.1+)
95% CI	(NE, NE)
Event-free rates^c^ at 9 months (%)	74.5
95% CI	(70.5, 89.4)

* NE* denotes not estimable.  + denotes censored observations

^a^One patient who died from complications of progressive disease before any postbaseline tumor assessments is included in this category

^b^One-sided *p*-value was based on exact test comparison of tislelizumab ORR versus reference rate (*H*_0_) of 0.35

^c^Event-free rates were estimated by Kaplan–Meier methodology with 95% confidence intervals estimated using Greenwood’s formula

^d^Medians were estimated by Kaplan–Meier methodology with 95% confidence intervals estimated using the Brookmeyer and Crowley method

**Fig. 3:**
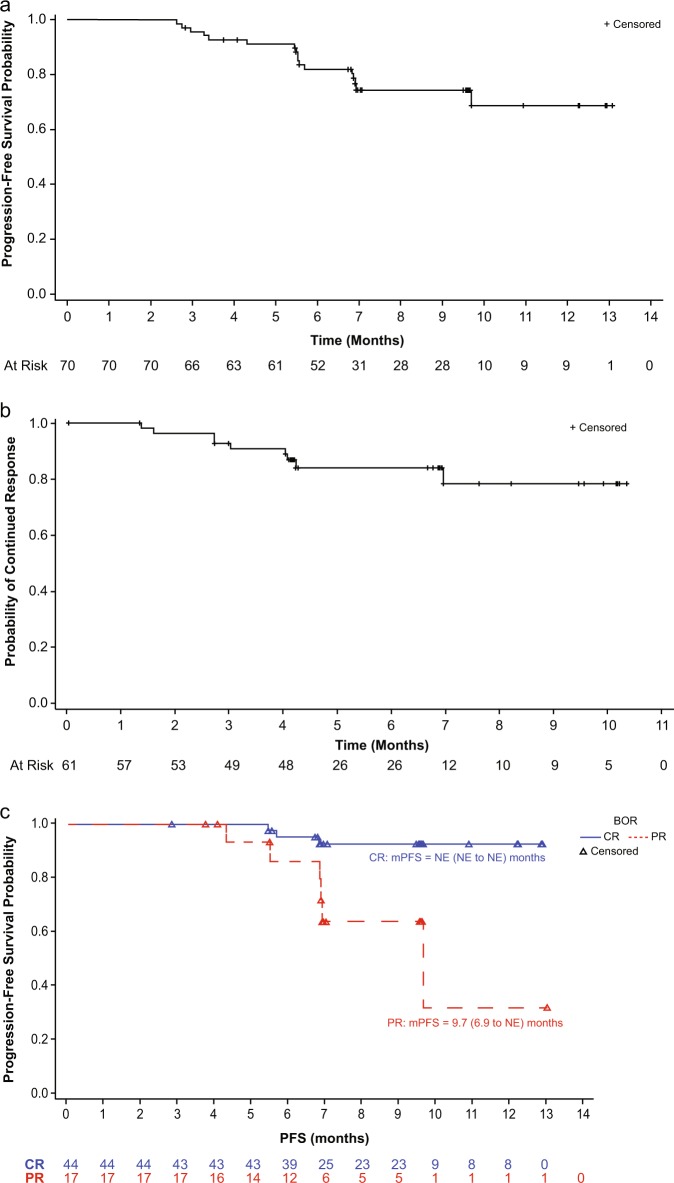
**a** Progression-free survival by the Independent Review Committee (IRC) per the Lugano classification. Kaplan–Meier plot for progression-free survival (PFS; shown as the percentage of patients alive without disease progression) for the 70 efficacy-evaluable patients. The median PFS was not reached after a median follow-up of 9.6 months. **b** Duration of response by IRC per the Lugano classification with objective response. Kaplan–Meier plot for DOR for all 61 patients who had a response. The median DOR was not reached after a median follow-up of 6.7 months after the initial response. **c** Progression-free survival by IRC per response category (complete response or partial response)

After a median follow-up of 9.6 months (range, 2.6, 13.1+), the median PFS has not been reached. At 9 months, the PFS rate was 74.5% (Table 2, Fig. 3a). Likewise, after a median follow-up from first response of 6.7 months (range, 4.2–6.9), the median DOR has not been reached for the 61 patients who achieved a response (Table 2, Fig. 3b, c). One patient died as of the data cutoff date due to disease progression, with a corresponding 9-month overall survival rate of 98.6%.

Almost all patients (92.9%) experienced at least one AE, with the majority of AEs being grade 1 or 2. Grade ≥ 3 AEs were reported in 21.4% of patients. Two patients experienced grade 4 events (increased serum creatine phosphokinase and thrombocytopenia), and there were no grade 5 events. Table 3 lists the treatment-emergent AEs reported in at least 5% of study patients. The most frequently reported AEs were pyrexia (54.3%), hypothyroidism (32.9%), weight gain (30%), upper respiratory tract infection (30%), leukopenia (18.6%), cough (17.1%), and pruritus (17.1%). The most common grade ≥3 AEs (each reported in two patients) were upper respiratory tract infection and pneumonitis. Eleven patients required at least one dose interruption or delay (dose reductions were not permitted) for management/resolution of AEs (Supplementary Table 1).

**Table 3:** Adverse events^a^

Term	All grades	Grade 3	Grade 4
	*n* (%)		
Patients with at least one adverse event	65 (92.9)	13 (18.6)	2 (2.9)
Hematologic events			
Leukopenia^b^	13 (18.6)	0	0
Neutropenia^c^	10 (14.3)	1 (1.4)	0
Thrombocytopenia^d^	8 (11.4)	0	1 (1.4)
Anemia	7 (10)	0	0
Nonhematologic events			
Pyrexia	38 (54.3)	0	0
Hypothyroidism	23 (32.9)	0	0
Weight gain	21 (30)	0	0
Upper respiratory tract infection	21 (30)	2 (2.9)	0
Cough	12 (17.1)	0	0
Pruritus	12 (17.1)	0	0
Rash	9 (12.9)	1 (1.4)	0
Alanine aminotransferase increased	9 (12.9)	0	0
Diarrhea	7 (10)	0	0
Aspartate aminotransferase increased	7 (10)	0	0
Hyperuricemia	6 (8.6)	0	0
Weight loss	6 (8.6)	0	0
Asthenia	5 (7.1)	0	0
Blood bilirubin increased	5 (7.1)	0	0
Headache	5 (7.1)	0	0
Hyperlipidemia	5 (7.1)	0	0
Pain in extremity	5 (7.1)	0	0
Chills	4 (5.7)	0	0
Influenza	4 (5.7)	0	0
Lung infection	4 (5.7)	0	0
Nausea	4 (5.7)	0	0
Back pain	4 (5.7)	1 (1.4)	0
Viral upper respiratory tract infection	4 (5.7)	0	0
Vomiting	4 (5.7)	0	0

^a^Data are for adverse events reported during treatment in the 70 patients included in the study. Listed events occurred in at least 5% of patients or two or more for grade 3 and any events for grade 4 on or before the data cutoff date of July 23, 2018

^b^Includes the MedDRA preferred terms, leukopenia and white blood cell count decreased

^c^Includes the MedDRA preferred terms, neutropenia and neutrophil count decreased

^d^Includes the MedDRA preferred terms, thrombocytopenia and platelet count decreased.

We evaluated the incidence and severity of AEs of special interest based on the known toxicity profile for anti-PD-1 therapies (Supplementary Table 2). These included infusion-related reactions, irAEs, and severe hypersensitivity/anaphylactic reactions and flu-like symptoms.

Infusion-related reactions were reported in 27 (38.6%) patients. The most frequently reported infusion-related signs and symptoms were pyrexia in 27 (38.6%) patients and chills in three (4.3%) patients; all were grade 1 or 2. All but four cases of pyrexia occurred on day 1 of study treatment and resolved within 24 h in response to either no treatment or antipyretics. Only one patient experienced a grade 3 infusion-related reaction (back and musculoskeletal chest pain) on study day 1, which resolved within 24 h.

Twenty-seven patients (38.6%) experienced an irAE (Supplementary Table 2). The most common of these included thyroid disorders (hypothyroidism [*n* = 14], hyperthyroidism [*n* = 2]), all grade 1 or 2, and one resulting in a dose delay. Notably, nine of the 13 patients had prior radiotherapy, a known risk factor for the development of thyroid dysfunction in patients with Hodgkin lymphoma [22]. Other irAEs included pneumonitis (*n* = 4; all in patients with prior bleomycin exposure), immune-related skin toxicities (*n* = 6; including dermatitis, erythema nodosum, pruritus, rash, and vitiligo), immune-related musculoskeletal events (*n* = 2; including osteoarthritis and blood phosphocreatine increase in a patient with concurrent hypothyroidism), immune-related liver injury (*n* = 1), and immune-related renal injury (*n* = 1). Four patients discontinued study therapy due to irAEs (three with pneumonitis and one with renal injury).

No patient experienced severe hypersensitivity/anaphylactic reaction, and four patients experienced grade 1 or 2 influenza during tislelizumab therapy.

## Discussion

Although most newly diagnosed cHL patients are likely to be cured of their disease, ~5–10% of patients will have primary refractory disease, and an additional 10–30% will relapse after having achieved a CR [1]. Treatment in first relapse or for primary refractory disease includes HDT/ASCT; however, the 5-year overall survival rate for patients with chemo-resistant disease at the time of HDT/ASCT is only 17% [23]. Therapeutic options for these patients include brentuximab vedotin [24] and anti-PD-1 therapy, both of which prolong survival. Longer term data with these agents suggest that a small minority of patients may achieve long-term disease-free survival without additional therapy (~10% for brentuximab vedotin at 5 years) [25, 26]; however, they are not expected to be curative in the majority of patients.

In a phase 2 study of 243 cHL patients treated with nivolumab who had previously failed both HDT/ASCT and brentuximab vedotin, the ORR was 69%, with 16% CRs [27]. An ORR of 87%, including 17% CRs, was observed in a separate study of 23 R/R cHL patients [10]. In a phase 2 multicohort study of 210 patients treated with pembrolizumab who had previously failed HDT/ASCT and brentuximab vedotin, or both, the ORR was 69%, with 22.4% CRs [11]. At 24 months follow up, 31% of patients remained progression free [28]. In a separate phase 1 study (*n* = 31), 65% achieved an objective response, including 16% CRs [9]. Based on these results, both nivolumab and pembrolizumab have received accelerated approval from the US FDA for the treatment of R/R cHL after three or more lines of therapy [29, 30]. Although achievement of CR correlated with improved outcome, CR was rare in both of these studies. Neither nivolumab nor pembrolizumab are approved for the treatment of cHL in China. More recently, the new PD-1 inhibitor sintilimab was approved for use in China based on phase 2 data demonstrating an objective response rate of 80% and a CR rate of 34% in Chinese patients with cHL [31].

In the current study of Chinese patients with R/R cHL, tislelizumab achieved a high level of overall response and CR as assessed by the IRC. Acknowledging differences in study design and patient population, the results reported herein compare positively with those for nivolumab, pembrolizumab, and sintilimab reported above, particularly the depth of response. Notably, CR rate does appear to correlate with DOR in patients in this trial and those treated with other PD-1 inhibitors, suggesting that increasing CR has potential to lead to better long-term outcomes [27, 32]. Although follow-up to date is short, few of the responders progressed during the course of follow-up. Rates of response were generally consistent across subgroups (Fig. 2), and the trend to achieve high response rates was observed even in those subgroups that have traditionally responded poorly to therapy, including heavily pretreated patients (≥3 prior lines of therapy) and those with refractory disease. Although only four patients had previously received brentuximab vedotin, all four achieved CR. Furthermore, studies of other PD-1 inhibitors have not shown a significant difference in response based on prior brentuximab vedotin treatment, supporting the notion that prior therapy is not a major determinant of response to immune checkpoint inhibition. These findings are encouraging and help to highlight the differentiated mechanism of action of anti-PD-1 versus cytotoxic therapies, as well as the potential differentiation of tislelizumab, compared with other PD-1 inhibitors, with its minimization of FcɤR binding on macrophages and abrogation of antibody-dependent phagocytosis-mediated effector T-cell clearance [8, 12].

A notable difference between this study and those evaluating nivolumab and pembrolizumab is the ethnic composition of the patient population, with this study enrolling solely Chinese patients. Underlying epidemiologic or genetic factors have the potential to impact responsiveness to PD-1 inhibition. Somatic alterations in MHC class I or II expression have shown correlation with response to PD-1 inhibition [33, 34], and underlying human leukocyte antigen variability has also been suggested to impact outcome [35]. Studies of other PD-1 inhibitors in patients with cHL in China [31] and Japan [36] have not shown the depth of responses observed in the current trial, however, arguing against race being a major determinant of response. Nonetheless, more in-depth studies of potential ethnic or geographic determinants of response are warranted.

Tislelizumab was generally well tolerated; the spectrum of tislelizumab-associated toxicities was similar to those reported among cHL patients treated with nivolumab [8] or pembrolizumab [11]. The type and severity of AEs reported in the current study are qualitatively similar to those reported in other series of both Chinese and non-Chinese tislelizumab-treated patients [14, 15], and the majority were mild or moderate in severity, manageable, and reversible. Key treatment-related toxicities generally fall into the categories of constitutional signs or symptoms (e.g., fatigue, asthenia, and headache), infusion-related AEs (e.g., pyrexia, chills, musculoskeletal pain, and cough), and irAEs (e.g., pneumonitis, dermatitis, and, thyroiditis). Most irAEs were either not treatment limiting or resulted in transient treatment delays, with four patients requiring discontinuation of tislelizumab for an irAE.

There are several limitations to our study. The major limitation is the single-arm design; with no other approved treatment options for this patient population in China, identifying a suitable control for a randomized study is challenging. In addition, relatively few patients had received HDT/ASCT or brentuximab vedotin prior to study entry, in contrast to studies of other anti-PD-1 therapies [8, 11]. Although fewer prior therapies could be suggestive of less-resistant disease, the relatively high proportion of patients with primary refractory disease (no response to any prior therapy; 25/70 patients) and maintenance of ORR at 80% in this population argues against this interpretation and suggests that prior therapies are not key drivers of the high response rates observed in this study. Finally, while the follow-up duration is relatively short, continuing longitudinal evaluation of this study population will further define the magnitude of treatment benefit.

In summary, this phase 2 study demonstrated high ORR and CR rates in R/R cHL patients treated with tislelizumab. The toxicity profile was consistent with that reported for anti-PD-1 therapies, with no new safety signals observed. Most toxicities were mild or moderate, manageable, and generally not treatment limiting. As such, tislelizumab conferred a favorable benefit versus risk profile and may represent an important new treatment option for patients with cHL.

## Supplementary information

Supplemental Material

## References

[1] Ansell SM (2012). Hodgkin lymphoma: 2012 update on diagnosis, risk-stratification, and management. Am J Hematol.

[2] Sun J, Yang Q, Lu Z, He M, Gao L, Zhu M (2012). Distribution of lymphoid neoplasms in China: analysis of 4,638 cases according to the World Health Organization classification. Am J Clin Pathol.

[3] Marafioti T, Hummel M, Foss HD, Laumen H, Korbjuhn P, Anagnostopoulos I (2000). Hodgkin and Reed-Sternberg cells represent an expansion of a single clone originating from a germinal center B-cell with functional immunoglobulin gene rearrangements but defective immunoglobulin transcription. Blood.

[4] Green MR, Monti S, Rodig SJ, Juszczynski P, Currie T, O’Donnell E (2010). Integrative analysis reveals selective 9p24.1 amplification, increased PD-1 ligand expression, and further induction via JAK2 in nodular sclerosing Hodgkin lymphoma and primary mediastinal large B-cell lymphoma. Blood.

[5] Roemer MG, Advani RH, Ligon AH, Natkunam Y, Redd RA, Homer H (2016). PD-L1 and PD-L2 genetic alterations define classical Hodgkin lymphoma and predict outcome. J Clin Oncol.

[6] Chen BJ, Chapuy B, Ouyang J, Sun HH, Roemer MGM, Xu ML (2013). PD-L1 expression is characteristic of a subset of aggressive B-cell lymphomas and virus-associated malignancies. Clin Cancer Res.

[7] Gopal AK, Chen R, Smith SE, Ansell SM, Rosenblatt JD, Savage KJ (2015). Durable remissions in a pivotal phase 2 study of brentuximab vedotin in relapsed or refractory Hodgkin lymphoma. Blood.

[8] Younes A, Santoro A, Shipp M, Zinzani PL, Timmerman JM, Ansell S (2016). Nivolumab for classical Hodgkin’s lymphoma after failure of both autologous stem-cell transplantation and brentuximab vedotin: a multicentre, multicohort, single-arm phase 2 trial. Lancet Oncol.

[9] Armand P, Shipp MA, Ribrag V, Michot JM, Zinzani PL, Kuruvilla J (2016). Programmed death-1 blockade with pembrolizumab in patients with classical Hodgkin lymphoma after brentuximab vedotin failure. J Clin Oncol.

[10] Ansell SM, Lesokhin AM, Borrello I, Halwani A, Scott EC, Gutierrez M (2015). PD-1 blockade with nivolumab in relapsed or refractory Hodgkin’s lymphoma. N Engl J Med.

[11] Chen R, Zinzani PL, Fanale MA, Armand P, Johnson NA, Brice P (2017). Phase II study of the efficacy and safety of pembrolizumab for relapsed/refractory classic Hodgkin lymphoma. J Clin Oncol.

[12] Dahan R, Sega E, Engelhardt J, Selby M, Korman AJ, Ravetch JV (2015). FcγRs modulate the anti-tumor activity of antibodies targeting the PD-1/PD-L1 axis. Cancer Cell.

[13] Desai J, Markman B, Sandhu S. Updated safety, efficacy, and pharmacokinetics (PK) results from the phase I study of BGB-A317, an anti-programmed death-1 (PD-1) mAb, in patients (pts) with advanced solid tumors. Presented at 31st Annual Conference of the Society for Immunotherapy of Cancer; 2016 Nov 9–13; National Harbor, MD.

[14] Desai J, Millward M, Chao Y, Gan H, Voskoboynik M, Markman B (2017). Preliminary results from subsets of patients (pts) with advanced gastric cancer (GC) and esophageal carcinoma (EC) in a dose-escalation/expansion study of BGB-A317, an anti-PD-1 monoclonal antibody (mAb). Ann Oncol.

[15] Horvath L, Desai J, Sandhu S, O’Donnell A, Hill AG, Deva S (2017). Preliminary results from a subset of patients (pts) with advanced head and neck squamous carcinoma (HNSCC) in a dose-escalation and dose-expansion study of BGB-A317, an anti-PD-1 monoclonal antibody (mAb). Ann Oncol.

[16] Desai J, Markman B, Sandhu SK, Gan HK, Friedlander M, Tran B (2016). A phase I dose-escalation study of BGB-A317, an anti-programmed death-1 (PD-1) mAb in patients with advanced solid tumors. J Clin Oncol.

[17] BeiGene. BeiGene presents preliminary phase 1 data for BGB-A317 in Chinese patients with advanced tumors at the 20th annual meeting of CSCO. 2017. http://ir.beigene.com/phoenix.zhtml?c=254246&p=irol-newsArticle&ID=2303469.

[18] Cheson BD, Fisher RI, Barrington SF, Cavalli F, Schwartz LH, Zucca E (2014). Recommendations for initial evaluation, staging, and response assessment of Hodgkin and non-Hodgkin lymphoma: the Lugano classification. J Clin Oncol.

[19] Cheson BD, Ansell S, Schwartz L, Gordon LI, Advani R, Jacene HA (2016). Refinement of the Lugano classification lymphoma response criteria in the era of immunomodulatory therapy. Blood.

[20] Brookmeyer R, Crowley J (1982). A confidence interval for the median survival time. Biometrics.

[21] Kalbfleisch JD, Prentice RL (1980). The statistical analysis of failure time data.

[22] Hancock SL, Cox RS, McDougall IR (1991). Thyroid diseases after treatment of Hodgkin’s disease. N Engl J Med.

[23] Stathis A, Younes A (2015). The new therapeutical scenario of Hodgkin lymphoma. Ann Oncol.

[24] Younes A, Connors JM, Park SI, Fanale M, O’Meara MM, Hunder NN (2013). Brentuximab vedotin combined with ABVD or AVD for patients with newly diagnosed Hodgkin’s lymphoma: a phase 1, open-label, dose-escalation study. Lancet Oncol.

[25] Chen R, Gopal AK, Smith SE, Ansell SM, Rosenblatt JD, Savage KJ (2016). Five-year survival and durability results of brentuximab vedotin in patients with relapsed or refractory Hodgkin lymphoma. Blood.

[26] Cohen JB, Kuruvilla J, Engert A, Ansell SM, Younes A, Lee HJ (2018). Nivolumab treatment beyond investigator-assessed progression: extended follow-up in patients with relapsed/refractory classical Hodgkin lymphoma from the phase 2 CheckMate 205 study. Blood.

[27] Armand P, Engert A, Younes A, Fanale M, Santoro A, Zinzani PL (2018). Nivolumab for relapsed/refractory classic Hodgkin lymphoma after failure of autologous hematopoietic cell transplantation: extended follow-up of the multicohort single-arm phase II CheckMate 205 trial. J Clin Oncol.

[28] Zinzani PL, Chen RW, Lee HJ, Armand P, Johnson NA, Brice P (2018). Two-year follow-up of keynote-087 study: pembrolizumab monotherapy in relapsed/refractory classic hodgkin lymphoma. Blood.

[29] Bristol-Myers Squibb Company. OPDIVO (nivolumab) injection, for intravenous use [prescribing information]. Princeton, NJ, USA: Bristol-Myers Squibb Company. https://packageinserts.bms.com/pi/pi_opdivo.pdf. Accessed 2 Mar 2017.

[30] Merck & Co, Inc. Keytruda® (pembrolizumab) for injection, for intravenous use [prescribing information]. Whitehouse Station, NJ, USA: Merck & Co, Inc. https://www.merck.com/product/usa/pi_circulars/k/keytruda/keytruda_pi.pdf. Accessed 2 Mar 2017.

[31] Shi Y, Su H, Song Y, Jiang W, Sun X, Qian W (2019). Safety and activity of sintilimab in patients with relapsed or refractory classical Hodgkin lymphoma (ORIENT-1): a multicentre, single-arm, phase 2 trial. Lancet Haematol.

[32] Manson G, Herbaux C, Brice P, Bouabdallah K, Stamatoullas A, Schiano J-M (2018). Prolonged remissions after anti-PD-1 discontinuation in patients with Hodgkin lymphoma. Blood.

[33] Roemer MGM, Redd RA, Cader FZ, Pak CJ, Abdelrahman S, Ouyang J (2018). Major histocompatibility complex class II and programmed death ligand 1 expression predict outcome after programmed death 1 blockade in classic Hodgkin lymphoma. J Clin Oncol.

[34] Roemer MGM, Advani RH, Redd RA, Pinkus GS, Natkunam Y, Ligon AH (2016). Classical Hodgkin lymphoma with reduced β2M/MHC class I expression is associated with inferior outcome independent of 9p24.1 status. Cancer Immunol Res.

[35] Chowell D, Morris LGT, Grigg CM, Weber JK, Samstein RM, Makarov V (2018). Patient HLA class I genotype influences cancer response to checkpoint blockade immunotherapy. Science.

[36] Maruyama D, Hatake K, Kinoshita T, Fukuhara N, Choi I, Taniwaki M (2017). Multicenter phase II study of nivolumab in Japanese patients with relapsed or refractory classical Hodgkin lymphoma. Cancer Sci.

