# Supplementary material for: Treatment of relapsed or refractory classical Hodgkin lymphoma with the anti-PD-1, tislelizumab: results of a phase 2, single-arm, multicenter study
Source: Leukemia. 2019 Sep 13;34(2):533–42. doi: 10.1038/s41375-019-0545-2 (PMC7214259; doi:10.1038/s41375-019-0545-2)
Supplement: Supplementary file 1 — Supplemental Material [file 41375_2019_545_MOESM1_ESM.docx]

***SUPPLEMENTARY MATERIAL***


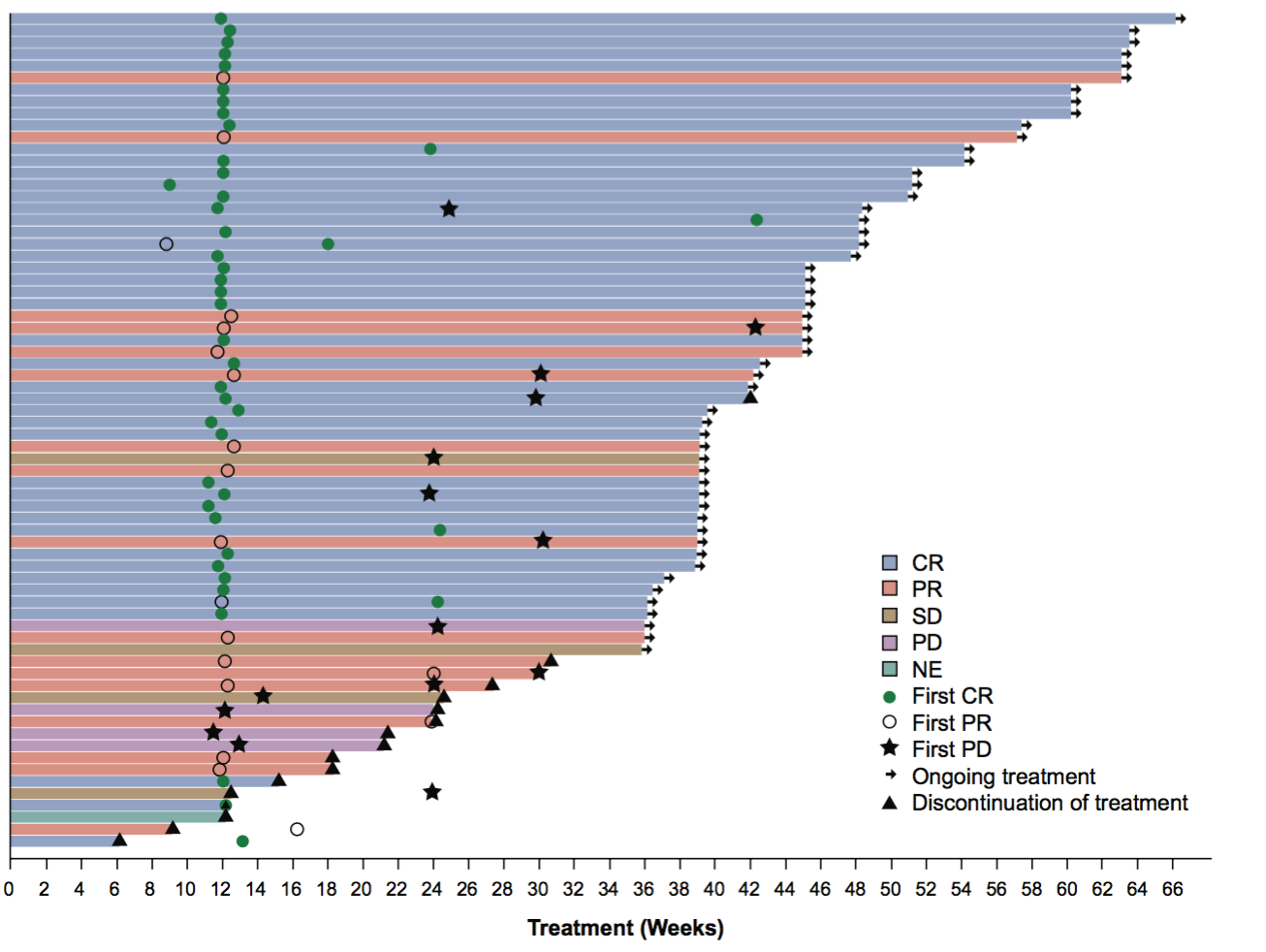


**Supplementary Figure 1. Duration of Treatment With Disease Response by IRC per the Lugano Classification for the Modified Safety Analysis Set**

Abbreviations: CR, complete response; PD, progressive disease; PR, partial response; NE, not evaluable; SD, stable disease

**Supplemental Table 1. Treatment-Emergent Adverse Events (TEAEs) Leading to Delay or Interruption of Tislelizumab***

| MedDRA Preferred Term | Tislelizumab  (N=70)  **n (%)** |
| --- | --- |
| Patients with at least one TEAE leading to dose modification | 11 (15.7) |
| Upper respiratory tract infection | 2 (2.9) |
| Asthenia | 1 (1.4) |
| Blood creatine phosphokinase increased | 1 (1.4) |
| Erythema nodosum | 1 (1.4) |
| Focal segmental glomerulosclerosis | 1 (1.4) |
| Headache | 1 (1.4) |
| Herpes zoster | 1 (1.4) |
| Hypothyroidism | 1 (1.4) |
| Interstitial lung disease | 1 (1.4) |
| Organizing pneumonia | 1 (1.4) |
| Osteoarthritis | 1 (1.4) |
| Pyrexia | 1 (1.4) |

*Patients with multiple events for a given preferred term were counted only once for that preferred term.

**Supplemental Table 2.** **Immune-Related Treatment-Emergent Adverse Events (TEAEs) by Category (Safety Analysis Set)***

|  | **Tislelizumab**  **(N=70)**  **n (%)** | | | | | |
| --- | --- | --- | --- | --- | --- | --- |
|  | **Maximum Severity** | | | | | |
| **Category^*^** | **Grade 1** | **Grade 2** | **Grade 3** | **Grade 4** | **Grade 5** | **All Grades** |
| Patients with at least one immune-related TEAE | 7 (10) | 14 (20) | 5 (7.1) | 1 (1.4) | 0 | 27 (38.6) |
| Thyroid disorder (hyperthyroidism, hypothyroidism) | 3 (4.3) | 12 (17.1) | 0 | 0 | 0 | 15 (21.4) |
| Skin adverse reactions (dermatitis, erythema nodosum, pruritus, rash, vitiligo) | 4 (5.7) | 1 (1.4) | 1 (1.4) | 0 | 0 | 6 (8.6) |
| Pneumonitis (interstitial lung disease, organizing pneumonia, pneumonitis) | 0 | 1 (1.4) | 3 (4.3) | 0 | 0 | 4 (5.7) |
| Musculoskeletal (blood creatine phosphokinase increased, osteoarthritis) | 0 | 1 (1.4) | 0 | 1 (1.4) | 0 | 2 (2.9) |
| Hepatitis (liver injury) | 1 (1.4) | 0 | 0 | 0 | 0 | 1 (1.4) |
| Nephritis and renal dysfunction (focal segmental glomerulosclerosis) | 0 | 0 | 1 (1.4) | 0 | 0 | 1 (1.4) |

* Patients with multiple events for a given category were counted only once for that category.
